# Is there a financial incentive to immigrate? Examining of the health worker salary gap between India and popular destination countries

**DOI:** 10.1186/s12960-017-0249-5

**Published:** 2017-10-19

**Authors:** Gavin George, Bruce Rhodes

**Affiliations:** 1 Health Economics and AIDS Division, University of KwaZulu-Natal, Durban, South Africa; 2 School of Accounting, Economics and Finance, University of KwaZulu-Natal, Durban, South Africa

**Keywords:** Human Resources for Health (HRH), Salaries, India, Migration, Purchasing power parity (PPP)

## Abstract

**Background:**

International migration is one of the factors resulting in the shortage of Human Resources for Health (HRH) in India. Literature suggests that migration is fuelled by the prospect of higher salaries available abroad. The extent of these salary differentials are unknown, and this study seeks to examine the salaries of selected HRH in India and four popular destination countries (United States of America, United Kingdom, Canada and the United Arab Emirates), whilst accounting for the in-country cost of living. This study will therefore determine truer financial incentives for Indian HRH to migrate abroad.

**Methods:**

A purchasing power parity (PPP) ratio is employed to equalise the international price of buying a representative basket of commonly bought goods (including food, entertainment, fuel and utilities). Using the PPP index, real differences in salaries are directly compared for selected work categories and different levels of work experience in the four respective countries.

**Results:**

Nurses in the USA can earn up to 82.7% more than their Indian counterparts. Nurses in Canada and the UAE reveal more modest salary differentials, yet still significant better off by up to 28 and 20% respectively. Only nurses in the UK are potentially materially worse off than nurses working in India. We observe significant potential PPP gains of up to 57.4, 99.1 and 94.4% for medical doctors in the USA, Canada and the UAE respectively. Medical specialists potentially experience the greatest income disparities with anaesthetists potentially earning up to 600% more than their counterparts in India. Radiologists operating in the UK and general surgeons working in the USA can potentially earn more than double that of their counterparts working in India. We observe more modest positive or negligible PPP gains in other selected countries for health specialists.

**Conclusion:**

Even when considering the differences in the cost of living, the financial incentive for selected cadres of Indian HRH to seek work abroad remains strong. The migration of Indian HRH to countries offering superior salaries makes it difficult for India to retain experienced health personal and compromises government efforts to render health care more accessible across the country.

## Background

The World Health Organization (WHO) emphasises the significance of a well-trained workforce, and having ‘the right staff in the right place’ if improved access to affordable health care and universal health coverage is to be achieved [1]. These goals are currently undermined by the shortage of health professionals, thereby contributing to the fragility of health systems in middle- and lower-income countries. This shortage of HRH is estimated to globally amount to 4.2 million—nurses, midwives and medical officers [1]. It is estimated that the basic healthcare systems of 57 countries are affected by the shortage of HRH and that about one third of these countries constitute emerging market economies [2]. These shortages compromise the capacity of the health system to deliver health care equitably, resulting in countrywide health disparities [3].

International migration has contributed to this shortage, particularly in middle- and lower-income countries [4]. Industrialised countries often become ‘recipient’ or ‘destination’ countries for qualified health personnel of ‘donor’ or ‘source’ countries which undermine the optimal functioning of health systems in these low- and middle-income countries [5]. India is one such country that has been affected by health worker migration. Universal and affordable health care has been the central objective of India’s health system since its independence. However, government efforts to create a network of public sector health facilities (i.e. primary health centres and sub-centres, community health centres, district hospitals and tertiary hospitals providing affordable services for all) have been unsuccessful in increasing access to health care in India [6]. This failure has, in part, been attributed to the continuous migration of skilled health personnel [6].

India has historically been the greatest exporter of health workers from the South Asian region [7]. Data from the All India Institute of Medical Sciences (AIIMS) reveals that 56% of all qualified medical doctors migrated abroad from 1956 to 1980. Recent data shows that about 75% of AIIMS graduates continue their studies in Western countries [7].

Whilst there are a number of factors which fuel international migration [1, 3, 8, 9], decisions to migrate are often family strategies with the goal of improving their economic circumstances [10]. Literature posits that the salaries of Indian HRH are dwarfed by salaries on offer abroad [11]. Identifying salary disparities through examining only exchange rates, limits our understanding of the true or real financial advantages of working abroad. The cost of living varies across countries requiring an analysis of economic indicators in order to fully determine the extent of the financial incentive to migrate. This study, therefore, seeks to evaluate salary differentials between selected health cadres in India and popular destination countries whilst accounting for the cost of living and inflation rates in these respective countries to determine the true disparities between HRH salaries. These results will reveal the extent of the financial pull to work abroad for Indian health personnel.

## Methods

To enable more informed comparisons on earnings, a purchasing power parity (PPP) index is required to equalise the cost of living across selected countries. Using a representative basket of commonly bought goods (including food, entertainment, fuel and utilities), the PPP is an exchange rate between two currencies that equalises the international price of buying that basket.

Our study uses a PPP ratio that converts the foreign salaries (expressed in their own national currency units) to the international dollar equivalent, published by the International Monetary Fund (IMF) [12]. An international dollar is a purely hypothetical currency where one international dollar (earned in another country) has the same purchasing power as one US dollar earned and spent in the USA. The PPP for the United States of America is set at 1.0 and acts as a baseline to compare all other countries. Examining the cost of living adjusted salaries for each of our selected countries determines the real financial incentives to emigrate within a given health profession.

The salaries are reported below in a series of tables for nurses, doctors and selected specialists respectively. The latter explores well-known areas of specialisation: radiographer, anaesthetist and general surgeon. These specialists all have an early career starting point and are often considered consultants as the years of experience increase. The lower and higher end estimates generally represent this often large difference. For the radiographer category, the data reported is for a diagnostic radiographer.

Each table presents the basic salary in its own national currency unit (NCU), the US dollar equivalent and then the PPP adjusted salary, also in US dollars. Finally, the percentage gaps between country PPP salaries, using the Indian figure as a base, are presented as a useful comparator. Both lower and upper bound percentage comparisons on the PPP result were made where possible.

The basic before tax salary is reported below. This would include any weightings based on location and other allowances or benefits. Some countries have more location-based salary variation than others. Whilst it is acknowledged that the ability to earn performance benefits will vary across countries and experience, these would only accrue to specific individuals under specific circumstances and are generally not considered. Whilst tax rates do vary across countries, preliminary post tax comparisons did not change the rankings significantly. One caveat is the case of Canada where an adjustment is made for overheads and is discussed more below.

### India

All government sector employees are paid according to guidelines laid down by the Indian Pay Commission which are adjusted every 10 years. The latest round of June of 2017, known as the 7th Pay Commission, recommended that all public sector salaries be raised by 14.5% and is widely expected to be implemented by the Government over the next 3 years [13, 14]. In order to maintain relevance, these increases have already been incorporated into the results.

Salaries in India generally vary by region and unique city classifications. X-cities are generally the wealthiest and command the higher salaries relative to Y-class and lastly Z-class cities. The upper X and lower Z class is reported below and used as an upper and lower bound respectively in the results tables [3, 15]. India has considerable regional salary variation where a state like Kerala offers the lowest and Delhi or Karnatake the highest [16].

As a government employee, a nurse requires a BSc with 6 months of experience or a diploma in general nursing with two and a half years of experience [3]. Government-employed nurses generally enjoy the highest salaries compared to their private counterparts [3]. Whilst the highest increases are to be found in the private sector, the relative levels remain in favour of government-employed nurses, consistent across all levels of experience [3].

Entry-level qualification for an MD, specialist and surgeon is a Bachelor of Medicine and Bachelor of Surgery (MBBS) and a post graduate diploma and 3 years relevant experience in a chosen specialty. The trend found for nurses is not quite the same for doctors where salary levels are very similar between the government and private sector, regardless of experience [3]. For radiographers, the entry requirement is Class 12 with science plus a diploma or a certificate in radiography with 1 year experience [3]. In this instance, the private sector is the worst payer especially in the early career stages of this specialty [3].

### United Kingdom

The UK data for nurses is based on the salary scale of its public sector health employer, the National Health Service. Scales were last revised in April 2017 under the ‘Agenda for Change’ and are based on a point system within different bands [8]. In addition, there is also a London weighting for those working in that part of the country [8]. Early career nurses are within band 4 [17]. Early career, or junior, doctors are paid both a basic salary and a banding supplement, which varies on the hours that they work [18]. In the UK, a junior doctor requires at least Foundation 1 and then Foundation 2 as a career starting point [19]. In some countries, a resident medical officer (RMO) is a junior doctor in training and in the UK the term RMO generally refers to a doctor in a private hospital [20].

In terms of the chosen specialists, anaesthetists and general surgeons generally receive the same level of pay. A radiographer in the UK needs a degree in diagnostic or therapeutic radiography. The selected path will determine whether a radiographer reaches the higher, experienced end of the salary scale [21]. An anaesthetist and surgeon in the NHS generally both require a 5-year degree in medicine, a 2-year foundation programme of general training, 6–8 years of specialist training and, in the case of surgeons, two additional years of core surgical training. The salaries are identical and are reported as such in the data [22, 23].

### United States

A registered nurse working in the USA requires an Associate of Science (ASN) in Nursing or a Bachelor of Science in Nursing (BSN) and a pass in the National Council Licensure Examination (NCLEX) [24, 25]. Salaries vary across the country and a national average is reported in the data. Prior to obtaining a licence, doctors in the USA are required to have a 4-year undergraduate degree, 4 years of medical school training, 3–7 years of residency training and pass the United States Medical Licensing Examination (USMLE) [26]. Radiographers require an associate’s degree in radiation science in addition to licencing which varies by state [27]. The anaesthetists and surgeons require a bachelor’s degree followed by graduate training that leads to a medical degree, followed by 4 years of residency training although the residency may be longer for surgeons [28, 29].

### Canada

Canadian provinces require a Bachelor’s degree in nursing which can be completed in 2 to 4 years [30]. As in the USA, there is some variation across the country and only provincial averages are reported in the data.

Doctors in Canada are required to go through the same educational process as found in the USA. After passing the Medical Council of Canada Evaluating Examinations (MCCEE—parts 1 and 2), the Medical Council of Canada awards a qualification known as Licentiate of the Medical Council of Canada (LMCC). This allows the graduate to practice medicine in Canada [31]. Radiographers require a 4-year degree in radiological technology that includes an annual summer practical experience in a hospital for 8 weeks [32]. To work in Quebec, a radiologist requires membership to the ‘Ordre des technologues en imagerie médicale et en radio-oncologie du Québec’ which is certified by the Canadian Medical Association (CMA) [33]. For specialists such as anaesthetists and surgeons, in addition to the medical training as a doctor, a person must register with the Royal College of Physicians and Surgeons of Canada (RCPSC) [34].

Depending on the province, around 47–75% of Canadian doctors and specialists are ‘incorporated’ private practitioners with their own assets, expenses, revenues and overheads [35, 36]. This is generally done for tax purposes but without taking this into account it may inflate the values reported below. Indeed, Petch et al. found that incorporating overhead charges ‘substantially’ affects physician income estimates and can be anywhere between 12.5% for emergency medicine and 42.5% for ophthalmology for those working in Ontario [37]. Overhead estimates do vary according by speciality and region [35] and as such an upper bound of 40% was chosen to scale down the upper bound of the salaries reported below. Thus, if a source for doctors or specialists reported a salary of 100,000 Canadian dollars as the upper bound, this was reported below as 60,000.

### United Arab Emirates

The vast majority of nurses in the UAE are migrants. Prospective nurses need to prove they have completed an accredited nursing or midwifery program with a minimum of 3 years training experience, have a basic life support certificate and a minimum of 2 years post registration experience [38]. Successful candidates must register with the appropriate local health authority. Foreign health workers are generally attracted by added benefits such as free accommodation, a travel and relocation allowance, health insurance, extensive paid leave and a tax-free income [38].

To work in the UAE as a radiographer, a Bachelor’s degree in radiotherapy is required and at least 2 years of experience [39]. Physicians must have graduated from a medical school listed in the Avicenna directory of medical schools published by the World Health Organization or the International Medical Education Directory of the Foundation for the Advancement of International Medical Education and Research. Local graduates in the UAE must have graduated from a university that is fully accredited by the Ministry of Higher Education and Scientific Research [39].

## Results

Tables 1, 2 and 3 illustrate that the different categories of Indian HRH workers can, in most cases, expect higher salaries in the selected destination countries. With regard to registered nurses (Table 1), the PPP results indicate that the USA is one of the most attractive countries in terms of higher earning potential. This is especially true for starter salaries where nurses in the USA can potentially earn 82.7% more in PPP terms compared to their Indian counterparts. These percentages do fall with increasing experience where the most experienced nurses in the USA earn 33.9% more than Indian nurses. The Canadian results, whilst also showing better PPP salaries with more experience are not as high as the USA. Canada sees better potential for mid and higher level experienced nurses where they could earn between 17.2% and 27.8% more than Indian nurses. Data indicates there is very little positive salary differentials between Indian nurse salaries and those offered in the UAE. Only very experienced nurses working in the UAE see a positive salary difference of around 20%. Indeed, the starting point and even mid-career salaries offered in the UAE are around 70% lower in PPP terms relative to India. Finally, the UK sees a rather flat if not slightly worsening salary position relative to Indian nurses insofar as it may be fair to suggest that, at all levels of experience, Indian nurses are materially better off staying in their home country.

**Table 1:** Registered nurse salaries from selected countries for three levels of work experience

		India^e^	UK	USA^i^	Canada^j^	UAE^k^
0–3 years	NCU^a^	727,904	21,909^f^	55,969	42,412	40,501
		856,524				175,970
	Rupee^b^	727,904	1,809,280	3,734,640	2,139,294	735,777
		856,524				3,196,827
	US ($)^c^	10,909	27,115	55,969	32,060	11,027
		12,836				47,909
	PPP ($)^d^	30,643	31,299	55,969	33,395	8347
		36,058				36,268
	% PPP ($) gap over India		2.1	82.7	9.0	− 72.8
			− 13.2	55.2	− 7.4	0.01
5–10 years	NCU	955,782	26,565^g^	63,249	65,325	59,573
		1,042,646	35,577			198,822
	Rupee	955,782	2,193,780	4,220,412	3,295,043	1,082,256
		1,042,646	2,938,006			3,611,977
	US ($)	14,324	32,877	63,249	49,381	16,219
		15,626	44,030			54,131
	PPP ($)	40,237	37,950	63,249	51,437	12,278
		43,893	50,827			16,569
	% PPP ($) gap over India		− 5.7	57.2	27.8	− 69.5
			15.8	44.1	17.2	− 62.3
10–20+ years	NCU	1,243,470	31,696^h^	70,116	78,000	39,732
		1,613,305	69,186			407,724
	Rupee	1,243,470	2,617,507	4,678,626	3,934,380	721,807
		1,613,305	5,713,491			7,407,076
	US ($)	18,635	39,227	70,116	58,962	10,817
		24,178	85,625			111,006
	PPP ($)	52,348	45,280	70,116	61,417	8189
		67,917	98,837			84,032
	% PPP ($) gap over India		− 13.5	33.9	17.3	− 84.4
			45.5	3.2	9.6	23.7

^a^Salary in own national currency unit. All at 2017 figures

^b^Author calculations. Indian Rupees using average 2017 to date market rate [44]

^c^Author calculations. US dollars using average 2017 to date market rate [44]

^d^Author calculations. International dollar (purchasing power parity, PPP) [12]

^e^X-cities (at 0 years) and Z-cities (at 3 years) and at 5–10 years and at 20–25 years [3]

^f^Corresponds to band 4, point 15 [8, 17]

^g^Band 6 [8, 17]

^h^Band 7 to 8c [8, 17]. Directors of nursing can earn 50% more than this [45]

^i^ Variation is large: US$41,000–US$70,000. (New York, California, Boston and Seattle can be 30–50% above national average. Indianapolis and St Louis are 8 and 14% below average respectively [46]

^j^Based on stated and averaged hourly rates at 1950 h per annum for nurses working in Ontario. Canadian nurses start at step 1, mid-career (5–10 years is step 3) with some regional variation especially due to remote region allowances [47]

^k^ Generally Dubai offer some of the higher rates and are used here [48] but there is some variation across the UAE but generally lower than those found in Dubai [49]

**Table 2:** Medical Doctor salaries from selected countries for three levels of work experience

		India^e^	UK^f^	USA^i^	Canada	UAE^l^
0–3 years	NCU^a^	1,177,807	26,614	78,057	57,888^j^	70,469
		1,464,684	30,805		155,910	581,748
	Rupee^b^	1,177,807	2,197,827	5,208,504	2,919,915	1,280,202
		1,464,684	2,543,926		7,864,221	10,568,551
	US ($)^c^	17,651	32,938	78,057	43,759	19,186
		21,950	38,124		117,857	158,385
	PPP ($)^d^	49,584	38,020	78,057	45,581	14,524
		61,661	44,007		122,764	119,899
	% PPP ($) gap over India		− 23.3	57.4	8.1	70.1
			− 28.6	26.6	99.1	94.4
5–10 years	NCU	1,439,512	36,461^g^	109,773	79,822^k^	82,212
		1,876,760	46,203		180,046	857,880
	Rupee	1,439,512	3,011,008	7,324,816	4,026,283	1,493,536
		1,876,760	3,815,518		9,081,670	15,585,010
	US ($)	21,573	45,124	109,773	60,340	22,383
		28,126	57,181		382,322	233,564
	PPP ($)	60,601	52,087	109,773	62,852	16,944
		79,008	66,004		141,769	176,810
	% PPP ($) gap over India		− 14.1	81.1	3.7	− 72.0
			− 16.5	38.9	79.4	123.8
10–20+ years	NCU	2,301,450	37,923^h^	145,039	87,804^k^	119,599
		2,878,530	70,718		290,304	855,826
	Rupee	2,301,450	3,131,742	9,678,008	4,428,902	2,172,742
		2,878,530	5,840,006		14,643,158	15,547,695
	US ($)	34,491	46,934	145,039	66,374	32,562
		43,139	87,521		219,449	233,005
	PPP ($)	96,887	54,176	145,039	69,137	24,649
		121,181	101,026		228,586	176,386
	% PPP ($) gap over India		− 44.1	49.7	− 28.6	− 74.5
			− 16.6	19.7	88.6	45.6

^a^Salary in own national currency unit

^b^Author calculations. Indian Rupees using average 2017 to date market rate [44]

^c^Author calculations. US dollars using average 2017 to date market rate [44]

^d^Author calculations. International dollar (purchasing power parity, PPP) [12]

^e^ [3]

^f^ Foundation years 1 and 2 respectively. In the UK, a medical officer is generally classed as a junior doctor [50]

^g^ Doctors continue to work and move onto specialities [50]

^h^Additions to basic salaries can be substantial through clinical excellence awards [51]

^i^ [52]

^j^The Canadian doctor starter salary is based upon 1–4 years of experience [53]. All Canadian salaries are scaled down by 40% [37]

^k^Additional years of experience could not be clearly identified so the same increases for the USA were used as a reasonable proxy [52] but it is acknowledged that some regional variation does apply to Canada [54]

^l^ For doctors, Abu Dhabi generally offers the highest salaries for the country [55]

**Table 3:** Specialist salaries from selected countries for three levels of work experience

		India	UK	USA	Canada	UAE
Radiologist	NCU^a^	482,769^e^	21,000^h^	30,181^k^	41,600^n^	12,293^q^
		969,385	68,000	75,641	66,000	243,416
	Rupee^b^	482,769	1,734,214	2,013,886	2,098,336	223,326
		969,385	5,615,549	5,047,292	3,329,091	4,422,111
	US ($)^c^	7235	25,990	30,181	31,447	3347
		14,528	84,157	75,641	49,891	66,272
	PPP ($)^d^	20,324	30,000	30,181	32,756	2534
		40,809	97,143	75,641	51,969	50,168
	% PPP ($) gap over India		47.6	48.5	61.2	− 87.5
			138.0	86.4	27.3	22.9
Anaesthetists	NCU	1,030,500^f^	26,350^i^	103,457^l^	97,394^o^	123,650^r^
		1,374,000	102,500	399,879	218,995	1,189,217
	Rupee	1,030,500	2,176,025	6,093,368	4,912,629	2,246,336
		1,374,000	8,464,615	26,682,700	11,044,259	21,604,372
	US ($)	15,444	32,611	103,457	73,623	33,665
		20,591	126,855	399,879	165,514	323,773
	PPP ($)	43,382	37,643	103,457	76,688	25,484
		57,843	146,429	399,879	172,406	99,101
	% PPP ($) gap over India		− 13.2	138.5	76.8	− 41.3
			153.1	591.3	198.1	71.3
General surgeon	NCU	924,015^g^	26,350^j^	116,186^m^	60,742^p^	12,293^s^
		3,435,000	102,500	393,981	218,954	243,416
	Rupee	924,015	2,176,025	7,752,736	3,063,873	1,139,772
		3,435,000	8,464,615	26,289,144	11,044,199	17,721,236
	US ($)	13,848	32,611	116,186	45,917	17,081
		51,478	126,855	393,981	165,513	265,578
	PPP ($)	38,899	37,643	116,186	47,828	12,293
		144,607	146,429	393,981	172,406	201,045
	% PPP ($) gap over India		− 3.2	198.7	23.0	− 68.4
			1.3	172.5	19.2	39.0

^a^Salary in own national currency unit

^b^Author calculations. Indian Rupees using average 2017 to date market rate [44]

^c^Author calculations. US dollars using average 2017 to date market rate [44]

^d^Author calculations. International dollar (purchasing power parity, PPP) [12]

^e^ [3]

^f^ [56]

^g^ From starting to 20+ years experience [57]

^h^ [21]

^i^ [22]

^j^ Same speciality level as anaesthetist

^k^ [58]

^l^ [59]

^m^ [60]

^n^ [58]

^o^ [61, 62]

^p^ [63]

^q^ [64]

^r^ [65]

^s^ [66]

Table 2 reports that newly trained Indian medical doctors might well be enticed by greater spending power in the USA, Canada and the UAE with PPP potential gains of 57.4, 99.1 and 94.4% respectively. The only exception at this career point is the UK where earnings potential is down by between 23.3 and 28.6%. There is a similar pattern for mid-career and experienced medical doctors. Similar patterns emerge in the UAE, but the salary ranges are large which may result in higher income earning potential than their counterparts working in India. Notable here is that Table 2 reports the Canadian salaries are discounted by 40% to account for the overhead estimate. Despite this, Canada remains a financially attractive prospect for Indian medical doctors.

Specialist salaries (Table 3) across all five countries report their national average over all three levels of experience. As such the PPP percentage gap does vary considerably. The USA offers much larger PPP adjusted salaries over their Indian counterparts, especially for anaesthetists who potentially earn nearly 600% more than their counterparts working in India. Relative to Indian earnings, this specialisation offers the highest PPP salaries across the selected destination countries including the UK (153% higher). Radiologists working in the UK see the next highest gains (138%) over their counterparts working in India. However the same cannot be said for general surgeons. Whilst the USA still offers some of the highest potential gains (172.5–198.7%), the other migrant countries are much lower where the UK is about parity (− 3.2 to 1.3%) to India. Canada and the UAE can potentially offer higher rates of 23 and 39% respectively but these gains are only realised at the upper end of our estimates.

## Discussion

Identifying salary differentials using PPP methodology is largely consistent with the literature that suggests health worker salaries in India are less lucrative than those achievable abroad [11]. HRH from India with varying levels of experience are economically better off when migrating, even though the extent of the financial improvement varies. In the context of a global shortage of HRH, the promise of better salaries in Western countries predominantly could potentially entice health professionals from India and other developing countries, leaving the health systems in these source countries compromised [40]. Furthermore, the draining of human health resources may dangerously perpetuate itself. With fewer HRH being available in contexts affected by health labour migration, patients face a strained health system whilst the remaining health professionals have a greater workload resulting in poor working conditions and additional motivation to migrate [41].

Strategies to get to the root causes of the phenomenon of Indian health worker migration have been prognosticated to be potentially daunting in scope and cost [42]. However, countries both sending and receiving health personnel could better manage the flow of health labour and its potentially negative consequences on the domestic health system of the source country [42]. The lure of comparably greater salaries could potentially be mitigated through a greater integration between health workforce planning, policy-making and efforts to strengthen health systems more generally. A comprehensive national policy for human resources is required to achieve universal health care with the public sector in need of designing appropriate packages of monetary and non-monetary incentives to encourage qualified health workers to work in rural and remote areas [9] rather than leaving it to privatisation to meet the health budget [7]. Policy makers should therefore also consider the importance of non-financial motivators such as working environment and skill development opportunities. Working conditions should be locally assessed and incentives should be managed to ensure health workers remain motivated [43].

This study has a number of limitations. The scope of this study is narrow in that it sets out to determine salary differentials between selected health cadres in India and their counterparts in popular destination countries sing the PPP methodology. This methodology does not account for the varying tax rates, which may further equalise the salaries across the countries analysed and, in certain circumstances, even result in poorer earning potential for health personnel working in India looking to migrate. It is, however, difficult to posit with any certainty that positive salary differentials will or have correlated with health worker migration patterns due to the lack of available data. Furthermore, the decision to migrate is often motivated by a number of factors, with earning potential, whilst important, only one consideration. Importantly, this study does not evaluate the demand and supply of the selected health cadres. Whilst the earning potential of anaesthetists, for example, appear significantly great than those of their counterparts working in India, the availability of these posts are not examined.

Future research should therefore examine which particular health cadres are in high demand abroad and examine HRH migration trends. This analysis will feed into the development and implementation of targeted interventions to reduce the loss of HRH and the associated impact on the domestic health system.

## Conclusions

The migration of Indian HRH to countries offering superior salaries could compromise India’s efforts to achieve universal health coverage. The potential for the majority of the selected health cadres working in India to earn higher salaries abroad is evident. However, salaries, whilst an important consideration, are only one of the factors influencing the migration of health workers. This study therefore adds to the body of literature identifying the earning potential of comparative employment abroad for HRH working in India.

## Abbreviations

AIIMS: All India Institute of Medical Sciences
ASN: Associate of Science
BSN: Bachelor of Science in Nursing
CMA: Canadian Medical Association
HRH: Human Resources for Health
IMF: International Monetary Fund
LMCC: Licentiate of the Medical Council of Canada
MBBS: Bachelor of Medicine and Bachelor of Surgery
MCCEE: Medical Council of Canada Evaluating Examinations
MD: Medical doctor
NCLEX: National Council Licensure Examination
NCU: National currency unit
NHS: National Health Service
PPP: Purchasing power parity
RCPSC: Royal College of Physicians and Surgeons of Canada
RMO: Resident medical officer
USMLE: United States Medical Licencing Examination
WHO: World Health Organization
